# Active Polymers Decorated with Major Acid Groups for Water Treatment: Potentials and Challenges

**DOI:** 10.3390/polym17010029

**Published:** 2024-12-26

**Authors:** Avneesh Kumar, Dong Wook Chang

**Affiliations:** Department of Industrial Chemistry and CECS Core Research Institute, Pukyong National University, Busan 48513, Republic of Korea; avneesh@pknu.ac.kr

**Keywords:** functional acidic polymers, metal complexations, porous nanostructures, membranes, water purification

## Abstract

Polymers exhibiting ion-conduction capabilities are essential components of water-purifying devices. These polymers not only transport selective ions but are also mechanically robust; thus, they can be processed as membranes. In this review, we highlight major acidic polymers and their engineered morphologies and optimized properties, including metal selectivity and water permeation or retention. Crucial phenomena, such as self-assembly in acid-group-functionalized polymers for driving water transportation, are discussed. It was observed that the phosphonic acid groups containing polymers are rather suitable for the selective adsorption of toxic metals, and thus, are superior to their sulfonated counterparts. Additionally, due to their amphoteric nature, phosphonated polymers displayed several modes of metal complexations, which makes them appropriate for eliminating a wide range of metals. Further observation indicates that aromatic-acid-functionalized polymers are more durable. Temperature- and pH-responsive polymers were also found to be promising candidates for a controlled water-treatment process. Nevertheless, considering the morphology, water retention, and metal adsorption, acid-functionalized polymers, especially phosphonated ones, have the potential to remain as the materials of choice after additional advancements. Further perspectives regarding improvements in acidic polymers and their fabricated membranes for water treatment are presented.

## 1. Introduction

Polymers and macromolecules are essential commodities in our daily life [1,2]. Their applications in households, energy sectors, and health restoration are now a reality, owing to their chemical and physical properties that can be modulated as needed [3,4,5,6]. In particular, polymers with ionic or charge centers have been successfully used in energy applications, such as batteries, and biomedical applications, such as drug delivery and blood filtration [7,8,9,10]. Ionic or charged polymers display various attributes necessary for their design and targeted applications [11]. These attributes are affected by ionic groups or charged centers that are capable of binding with other external molecular species or ions and allow for long-range charge transportation within macromolecules [12]. The charge transportation and morphology evolution in these ionic polymers can extend the utilization of such polymers in filtration devices, in which a certain type of molecule or species is selectively filtered to purify the original fluid [13]. In these devices, filtration usually occurs through a membrane composed of a functional polymer with a specific morphology suitable for the process and efficiency [14]. To achieve a robust and efficient membrane that can filter a fluid or liquid with a degree of selectivity toward the analytes, the relevant polymer must contain a unique set of ionic groups that not only participate in the filtration process but also create various features within the membrane to ensure that the membrane works efficiently and selectively [15,16]. These features include the porosity, thickness, high reflux, and selectivity toward certain toxic metals. Methods for obtaining clean water include wastewater purification, seawater desalination, and atmospheric-water-molecule harvesting. In these water-purification processes, a suitable “membrane material” is the main and essential component through which filtration occurs. Challenges such as high energy consumption or low water production associated with traditional water-purification technologies can be mitigated by using functional polymers, including ionic or responsive polymers.

**Figure 1:**
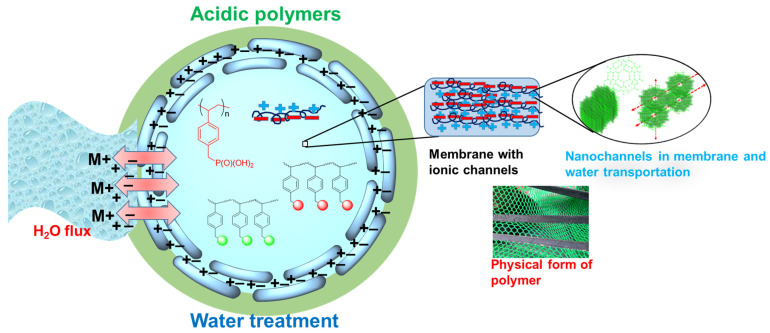
Ionic polymers as a medium (beads or membrane) in which they can acquire a suitable superstructure that can facilitate a favorable water flux in which the toxic metal ions from the water are adsorbed by the medium; thus, the water is purified. These polymeric membranes have ionic channels and suitable thermal/mechanical properties for long-term usage.

At present, membranes fabricated from related ionic polymers are being integrated into water-filtering devices in which a narrow spectrum of metals is eliminated or reduced to a certain level to make the water drinkable. For water filtration, the membrane must be stable, highly efficient, durable, and cost-effective, similar to its polymer precursor. The polymer precursor of a membrane is composed of multiple types of monomers decorated with functional groups. In this review, we emphasize the importance of polymers containing major acidic groups as filter components for water treatment or purification [17]. Polymer-based polyelectrolytes, along with their advantages and disadvantages, are discussed. Figure 1 displays a summary of the current review focusing on acidic polymers or polyelectrolytes and their superstructures containing nanochannels for a selective and efficient water flux. These acidic groups in a polymer fabricated as a membrane or as an assembly of beads interact with the toxic metal ions in water and eliminate these ions via complexation or adsorption. Furthermore, the focus of our review is the future developments in these acid-group-functionalized polymers for water treatment. Additionally, this review provides a concise description of polymers bearing major acid groups so that up-to-date and concrete knowledge on these materials can be obtained and applied when designing more robust acidic polymer materials for similar applications in the future. Furthermore, this review highlights the prevalent challenges associated with the efficiency of these polymers and offers a roadmap for solutions. The possibility of using acid-functionalized biomaterials as environmentally friendly systems for water-purification devices is also discussed.

## 2. Acid-Functionalized Polymers

**Figure 2:**
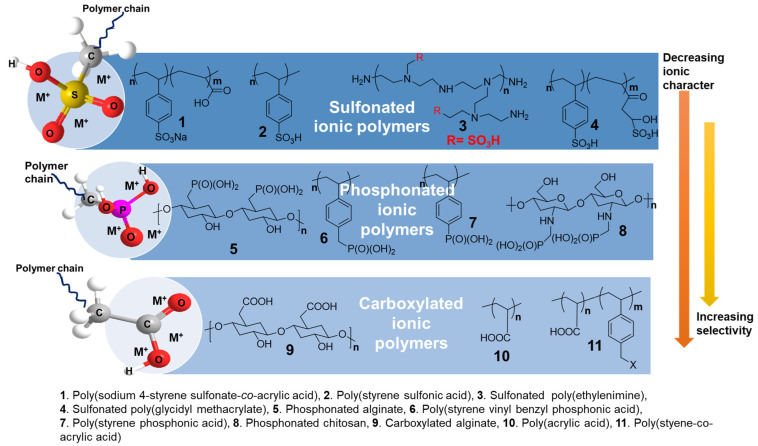
Representative examples of synthetic and bio-based sulfonated, phosphonated, and carboxylated polymers used as ionic or acidic precursor to fabricate their products for water treatment. Among these functional polymers, the sulfonated derivatives display a higher ionic dissociation but lower selectivity toward a specific metal. The water retention power of each polymer is directly related to the nature of the acidic groups.

Generally, acidic polymers are categorized into polyanions and polycations, depending on the nature of the acid group. These groups may be present as a pendant in the side chain or linked to the main polymer chain [18,19,20]. Acidic groups, such as sulfonic, phosphonic, and carboxylic groups, were introduced into a number of polymers, depending on their pH, for the removal of metals from water. Figure 2 shows a few representatives of sulfonated, phosphonated, and carboxylated ionic polymers, along with an overview of their building blocks, backbones, and properties, such as their ionic character and selectivity toward a metal.

Acidic functionalities are needed in polymers for metal complexation to achieve a binding strength and sorption ability. Sulfonic-acid-containing polymers have a higher metal retention capacity and are superior to all other analogs; thus, they are suitable for water treatment (Figure 2). Other polymeric derivatives based on carboxylic and phosphonic acids were successfully explored for selective metal retention. In sulfonated polymers, complexation with metals is usually dominated by electrostatic forces; therefore, these polymers act as polyelectrolytes in water and aqueous solutions. In phosphonated derivatives, metal binding involves both electrostatic and covalent interactions. Table 1 presents an overview of some acidic and polyelectrolyte polymers and their properties.

Polymers with specific acidic groups are susceptible and selective toward a set of metallic ions for water treatment and the solid-phase extraction of heavy metals [33]. Several chemically different monomers, namely, vinyl derivatives, functionalized acrylic, styrene, iminodiacetic, and aromatic-substituted ones, were used as building blocks to produce these polymers via radical, controlled radical, and metal-mediated polymerization methods [34,35,36,37,38]. Furthermore, dissociation of the acidic group at a certain pH and the effect of the substituent moiety on the ionic nature of the corresponding monomer significantly affect the performance of these polymers. The acidities of these ionic groups decrease in the following order: –SO_3_H > –P(O)(OH)_2_> –COOH. The dissociation constants of these groups also differ and can fluctuate at a given pH; this enables metal binding with selectivity [39,40]. Polymers produced via polymerization can be processed into various physical forms, including hydrogels, beads, fibers, sponges, and membranes [41,42,43,44]. The morphologies of these polymeric products play a crucial role in their application in water treatment and toxic metal removal. Other parameters, such as the porosity, specific surface area, sorption kinetics, and mechanical and thermal properties, are also correlated and modulated to improve the performance of the products [45,46,47,48]. For commercial purposes, the cost-effectiveness and durability of a fabricated polymeric product are also considered when projecting technology. During the removal of metals from polluted water, ion exchange and complexation are the predominant mechanisms, mostly affected by the strength of the acidic groups in the polymer. The hydration of acidic groups also influences the ion exchange and complexation [49]. In a strong acid group, such as the –SO_3_^−^ group, ion exchange can occur rapidly. Additionally, a proton/electron donor site in a relevant functional group can significantly increase or decrease the metal affinity, thus affecting the efficiency of the polymer for water filtration. To design a material with selectivity toward a certain metal ion, the electronegativity, valence, size, charge, and polarity of the metal must be determined. A suitable acidic group that follows the hard and soft acid and base theory is introduced into the polymer structure [50,51]. The nature of interactions between the acidic group and a metal ion is dependent on hard–hard and soft–soft combinations that lead to ionic interactions and coordination bonds, respectively. Selectivity and performance can be further controlled by introducing other chemical segments into the polymer backbone or side chains. In some cases, the ionic polymers for water purification are made water-soluble and -insoluble for recycling.

As described in Table 2, the bio-based polyelectrolytes have been successfully created to minimize environmental impact [52,53]. Environmentally friendly polymers include alginates, poly-γ-glutamic acid, chitosan, and their derivatives based on sulfonic, carboxylic, and phosphonic acid functionalities. These biopolymers are nontoxic and biodegradable; however, their poor performance should be improved by tuning their chemical and physical properties. A few of these functionalized acidic bio-based polymers, along with their properties and applications, are summarized in Table 2.

The bio-based polymers have certain limitations with regard to their chemical functionalization and the properties thereof. As these polymers are mostly based on sugar or amino acid building blocks, their long-term stability and function are still in question. One of the peculiar features of these bio-based polymers is their water solubility, which can allow users to recycle them. Also, it is challenging to pulverize a biopolymer into its fine powder form and to eliminate further impurities. However, these biopolymers can be combined with the synthetic counterparts to accomplish the desired properties and tune their performances. Biopolymers are also very expensive to produce as compared with the traditional fossil fuel-based materials. Another issue with biopolymers could be their complex structure, which can hinder processing these materials into a membrane or film, thereby limiting their usage for water-treatment devices. Although biopolymers originate from their respective renewable resources, their production must be managed in a sustainable manner, which includes cultivating the crop for their production in such a manner that it neither affects the food production nor causes deforestation.

**Figure 3:**
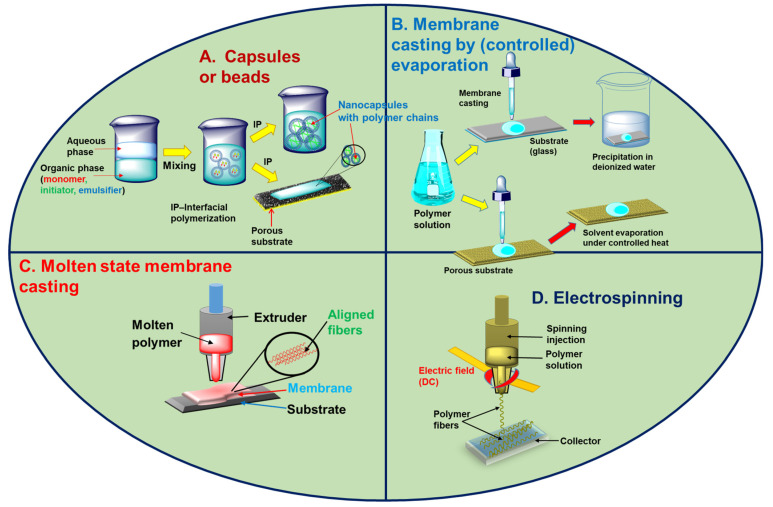
Widely used methods for casting membranes from respective polymeric precursors. Few processes are implemented to form beads or capsules of the ionic polymer. (**A**) A polymeric membrane can be fabricated by polymerizing (interfacial polymerization) the monomer at the interface of the organic–aqueous phase in which the nanocapsules with aligned polymer chains are deposited on a porous substrate. (**B**) Polymeric solution is deposited on a substrate, and a free-standing film or membrane can be obtained by evaporating the solvent either via precipitation in a non-solvent (deionized water) or under controlled heat. (**C**) Industrial methods, such as solvent-free extrusion of a polymer in its molten state, can also generate aligned fibers in a membrane. (**D**) An advanced version of extrusion involves applying an electric field (high-voltage DC) via which the fibers of the polymer can be produced on a substrate to cast a membrane as the final product.

Several methods are employed to fabricate ionic polymers into a targeted physical form or product. Figure 3 displays the common techniques used in academia and industry for fabricating membranes or other products from ionic or acidic polymer precursors. For bead formation, a typical slow solvent evaporation method can be employed with an appropriate polymer precursor or functionalized monomer building blocks polymerized during solvent evaporation (Figure 3A,B) [64,65]. Beads with different sizes and shapes can be obtained by changing the concentration of the polymer and selecting a suitable solvent. The beads are assembled in a water-treatment device for various applications. To prepare polymeric nanoparticles, traditional self-assembly of amphiphilic block copolymers, self-crosslinking of single-chain macromolecules, nanoprecipitation, or emulsion–miniemulsion polymerization methods were adopted [66]. In the most common water-treatment devices, acidic polymers are fabricated as membranes for filtration.

The membranes cast from acidic polymers are classified as (1) isotropic or symmetric and (2) anisotropic or asymmetric, depending on the nonporosity and charged or uncharged polymeric chains [67,68]. Isotropic or symmetric membranes are manufactured by using charged and noncharged polymeric materials in which the porosity and pore size are modulated. Usually, isotropically charged or ionic membranes are integrated for electrodialysis or separation processes, where the performance is affected by ionic interactions between the polar groups of polymers and oppositely charged impurity molecules. By contrast, the performance of anisotropic or asymmetric membranes is directly related to the structural or chemical heterogeneity of their polymeric networks. Several techniques were developed for fabricating polymer-based membranes (Figure 3). Interfacial polymerization (IP), sputtering, solution casting, extrusion, melt pressing, phase inversion, and electrospinning were used in both academia and industry [69,70,71,72,73,74]. The selection of the technique is based on the physical and chemical properties of the polymer considered for membrane production. These fabrication techniques can be further optimized with respect to parameters such as the choice of solvent (polar/nonpolar), temperature variation, substrate selection, and utilization of electrostatic forces. Phase inversion is used to convert a solid polymer into a solution or molten state by using different parameters, such as selecting an appropriate solvent and controlling the melting temperature [70,71,72]. Commercially or on a large scale, membranes are manufactured by casting a polymeric solution (with additives) onto a suitable glass substrate. The polymer solution deposited on the glass is then immersed in a non-solvent agent, such as pure deionized water (Figure 3B). This immersion–precipitation method is used to fabricate membranes with high tensile strength and porosity and improved hydrophilicity. Owing to the formation of small-diameter pores during the process, this method can increase the thermal shock resistance and volumetric power density of the membrane. In the subsequent modification step, evaporation and precipitation can be controlled by regulating the speed and temperature to cast a more homogenous membrane (Figure 3B). A low or high temperature at which the polymer solution is evaporated can have a substantial impact on the morphology of the membrane because the dynamics of the polymer chains directly influences the final shape and structure of the membranes. In such a thermally induced phase transformation of separation, a membrane with a high porosity and few defects can be obtained [75]. In the induced-phase approach, an ionic monomer is polymerized at the interface between water and a suitable organic solvent in a reaction reservoir, yielding an ultrathin membrane that can be applied in the ultrafiltration and reverse osmosis of water (Figure 3A). In the induced-phase process, polymers can also be produced as capsules or beads. More advanced techniques for fabricating polymeric membranes include polymer extrusion under cold and hot conditions without the use of harmful solvents [76]. During extrusion, the polymer is extruded at its melting point through a predesigned piston–cylinder assembly, which produces aligned polymer fibers (Figure 3C). Owing to the unique alignment of the polymer fibers in the membrane, the mechanical properties are improved, and the pore size and thickness are controllable. A modified and extended version of extrusion, known as electrospinning, is also implemented for fiber formation under an electric field, thereby replacing the need to heat the polymeric solution (Figure 3D). During electrospinning, a viscous polymer solution flows through an injector and a needle under an electric field [77]. When the electric field surpasses the surface tension, the polymeric solution flows out as fibers that accumulate on the collector.

Because the aim of this review was to present the most recent and key acidic-polymer-based membranes or similar systems, the following sections provide a comprehensive discussion of the main acidic polymeric materials and their related performances, including their distinctive features and future prospects.

## 3. Polymers with Sulfonic Acid Groups for Water Purification

Since the discovery of Nafion, a sulfonated and fluorinated polyethylene derivative, aromatic and aliphatic polymers with –SO_3_H groups have been used as ion-transport materials in fuel-cell batteries [78,79]. Sulfonic acid groups are typically introduced into a polymer via a functionalized monomer route or post-modification of the polymer in successive sulfonation steps [78,79]. The degree of sulfonation or concentration of sulfonic acid groups within a polymer affects the mechanical strength, morphology, crystallinity, and water uptake of the relevant polymer. In water-treatment membranes, sulfonic acid groups play a significant role in water retention and metal complexation. The presence of other chemical substituents, such as alkyl chains and aromatic rings, can also impact the metal coordination and water-purification efficiency of the polymeric membrane. In addition to the water filtration ability of sulfonated membranes for the removal of certain metal ions, the coordination chemistry of the ionic groups (–SO_3_H) must be understood. According to previous studies, sulfonate anions weakly coordinate with most transition-metal complexes [80]. The weak interactions of sulfonate anions with the metals are caused by the formation of the hydration sphere, in which the solvent (water) molecules are not easily displaced by anions [81,82]. These weak interactions between the sulfonate anions and the metal further affect the complexation and decomplexation processes during water purification. Note that the oxygen atom in a sulfonate anion can simultaneously form a bridge with two/three metal centers, which, in turn, can efficiently remove the metal from the water. In polymers, multiple sulfonate anions act cooperatively to provide a stable structure to the membrane and effectively facilitate the elimination of large concentrations of metal ions.

**Figure 4:**
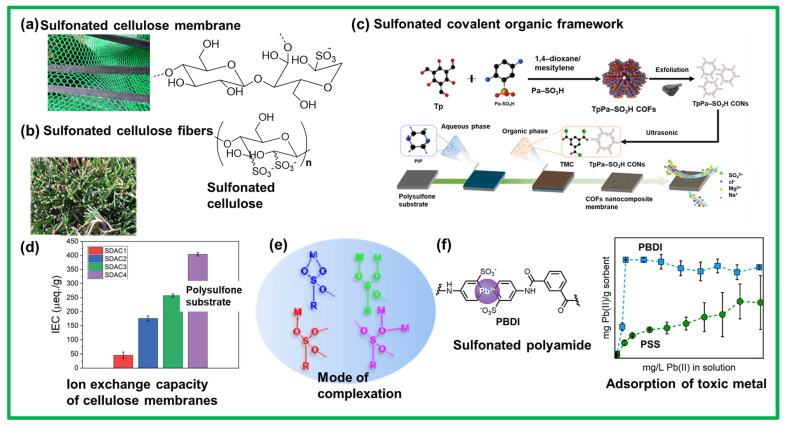
Biomaterial- and artificial-polymer-based sulfonated membranes and fibers for water treatment. (**a**,**b**) Artistic view of a membrane and fibers made up of sulfonated cellulose producing a porous structure through which water can pass and the metallic ions can chelate with the sulfonic acid groups. (**c**) Polymeric covalent organic frameworks fabricated as a membrane for water purification. (**d**) Ion-exchange performance of a sulfonated cellulose membrane. (**e**) Different modes of metal-ion chelation with the sulfonic acid group. (**f**) Membrane based on high-performance polyamide and metal adsorption from polluted water. Adapted with permission from *Adv. Sustainable Syst.* [91], Copyright 2022 Wiley-VCH GmbH. This is an open access article. Reprinted with permission from *npj Clean Water* [94], Copyright 2023 Nature Portfolio. This article is licensed under a Creative Commons Attribution 4.0 International License, which permits use, sharing, adaptation, distribution, and reproduction in any medium or format, as long as you give appropriate credit to the original author(s) and the source, provide a link to the Creative Commons license, and indicate whether changes were made. Adapted with permission from *ACS Appl. Polym. Mater.* [96], Copyright 2022 American Chemical Society.

Various polymers, such as aromatic and nonaromatic polymers, were developed with different degrees of sulfonation to investigate the water retention and metal adsorption in a series of membranes or other similar systems based on sulfonic acid groups. Pristine and blended polymeric systems, such as sulfonated polysulfone, sulfonated poly(ether sulfone), sulfonated polyphenylene, and sulfonated polyvinylidene fluoride, were widely processed as membranes and displayed improved charge characteristics, permeability, thermal/chemical/mechanical resistance, hydrophilicity, and separation performance [83,84,85,86]. However, the degree of sulfonation or number of sulfonic acid groups within the membrane material can directly affect the swelling and tensile strength, resulting in poor efficiency. Recently, more emphasis has been placed on producing materials in a green manner; thus, attention has shifted toward bio-based and renewable materials to reduce the production costs, improve the functionality, and minimize the environmental impact. Cellulose, a polysaccharide, has become the material of choice for manufacturing ion-selective membranes because of its abundance, low cost, and nanostructure [87,88]. The properties of cellulose can be optimized by changing the chain length or varying the number of glucose units. For the required application, cellulose can be crosslinked via additives or direct chemical modification of the precursor [89]. Figure 4 depicts selective examples of polymeric materials, including bio-based materials (cellulose) and a special class of polymers commonly known as covalent organic frameworks (COFs) decorated with sulfonic acid groups, showing efficiency in eliminating toxic metals from water. Figure 4a,b present artistic views of sulfonated cellulose membrane and fibers with porous and interconnected domains, respectively, along with two different chemical structures. Usually, the pristine cellulose fibers are functionalized with sulfonic acid groups via two steps: (1) periodate oxidation and (2) sulfonation [90,91].

The highly reactive dialdehyde is an intermediate product that forms after oxidation and offers possibilities to functionalize the cellulose surface with carboxylic acids and imines. The dialdehyde cellulose derivative is converted into sulfonated cellulose via treatment with sodium bisulfite in deionized water. The degree of sulfonation can be determined by measuring any residual aldehyde and then titrating with sodium hydroxide [91]. Several studies were conducted to establish the correlation between the water uptake and the degree of sulfonation in these membranes [92]. Achieving a certain molecular weight and optimal degree of sulfonation is crucial in commercial membranes. Investigations of the physicochemical and ion-transport properties of these sulfonated cellulose membranes/fibers have indicated that with an increasing degree of sulfonation (SDAC4 1.30), both the water uptake and ion-exchange capacity significantly increase (182% and 404 µeq. g^−1^, respectively), as required for easy water influx and metal complexation (Figure 4d,e) [91]. After these sulfonated cellulose membranes were tested for the selectivity and adsorption of metals found in polluted water, these membranes were found to be suitable for achieving promising results in water treatment. The selectivity and adsorption of metal ions on the surfaces of these membranes can also be tuned by adjusting the pH and loading temperature. These sulfonated cellulose membranes were found to be effective toward Fe^3+^, Pb^2+^, and Cu^2+^ with excellent selectivity and high efficiency. Additionally, because of their bio-based origins, cellulose-based acidic membranes can be considered as green and renewable adsorbents. Further studies on the adsorption mechanism revealed multiple modes of metal complexation via chelation with sulfonic acid groups within the membrane (Figure 4e) [93]. As a hard monoacid, sulfonic acid (R-SO_3_H) adsorbs metallic ions via an ion-exchange process that predominantly involves electrostatic interactions. The high acidity of the sulfonic acid groups further validates the strong adsorption of metallic cations.

Recently, a special class of polymers with a predesigned preprogramed morphology and degree of order was introduced as COFs [94]. In these COFs, the molecular design principles and interactions are exclusively affected by a predesigned monomer that is polymerized entirely in a controlled manner with precision [94]. COFs are porous crystalline materials in which the unique topology of the monomer offers a confined molecular space and an engineered interface to study various charge dynamics. Owing to their unique and distinct features, COFs were also used for water-treatment assemblies after processing. In this regard, sulfonated COF nanosheets (S-CONs) with improved water permeation and nanofiltration abilities were proposed [94]. As shown in Figure 4c, a sulfonated amine monomer (Pa-SO_3_H) was polymerized to produce a network of S-CONs. These S-CONs with a uniform distribution of –SO_3_H groups were integrated within the layers of polyamide (PA) to create nanochannels that exhibited efficient separation performance, long-lasting stability, and anti-fouling performance. These sulfonated COFs were successfully fabricated as thin and stable membranes that displayed a high water permeance and effective nanofiltration after treatment with impure water (Figure 4c) [95].

Commercially available poly(sodium4-styrenesulfonate) (PSS) is a global polyelectrolyte candidate for removing heavy metals from water. The crosslinked form of PSS is not water-soluble, whereas the counterpart crosslinked form is soluble in water, and thus, remains effective for metal removal. Engineering plastics or high-performance artificial polymers, namely, polyetheretherketone, polyethersulfone, PA, and their copolymer derivatives, were also considered for similar purposes owing to their suitable thermal, chemical, and physical properties; thus, researchers can process them in the membrane form. Both sulfonated polyetheretherketone and sulfonated polyethersulfone were extensively tested as membranes for the removal of metals, such as Pb, Hg, Ni, and Cr, from wastewater [96]. In 2023, an IP procedure was implemented to generate PA with sulfonic acid groups, which resulted in a semi rigid poly(2,2′-disulfonyl-4,4′-benzidine isophthalamide) (PBDI) (Figure 4f) [96]. As mentioned earlier, owing to the strongly acidic nature of sulfonic acid groups, their functionalized polymers or membranes are capable of binding with a wide range of metals. Similarly, sulfonated PBDI demonstrated a superior Pb(II) removal capacity of approximately 410 mg/g compared with PSS (260 mg/g); this was attributed to the location of the sulfonic acid groups and the rigid PBDI backbone facilitating coordination with Pb(II) without any precipitation (Figure 4f, right side) [97]. A comparative study and coordination models of Pb(II) with the sulfonic acid groups of PBDI and PSS indicate a similar hypothesis [97].

Considering the coordination chemistry of sulfonic acid group, it was established that the sulfonated ligands display poor “non-coordinating” anions. When in water, these ligands show weaker displacement for water molecules present inside the coordination sphere of the metal ion, and hence, affect the adsorption process. The main advantage that sulfonated groups can offer is their ability to undergo cooperative bonding effects with the metals, thereby allowing for the adsorption of a high concentration of metal ions. In a polymer, several factors, such as the chain dynamics and morphology, can play crucial roles and determine the effectiveness of the sulfonated ligand linked to the polymer chain or side chain. Also, the location and number of the sulfonated ligands or monomers directly influence the porous architecture of the polymer before and after fabrication. In a polymeric network, all acidic groups may not be accessible to the metallic ions upon water treatment, which, in turn, can affect the efficiency of the membrane. A large number of acidic groups at a local level in a polymeric membrane may also change the degree of porosity. If the porosity collapses upon water treatment, the material may no longer be as effective as it was in a previous water-purifying cycle. Therefore, a high level of precision for decorating these sulfonic acid groups in a polymer chain remains a focus of research. Given all these aspects, obviously the sulfonated polymers evaluated here thus far have variations in their properties, as is required for water treatment.

## 4. Polymers Functionalized with Phosphonic Acid Groups for Water Purification

**Figure 5:**
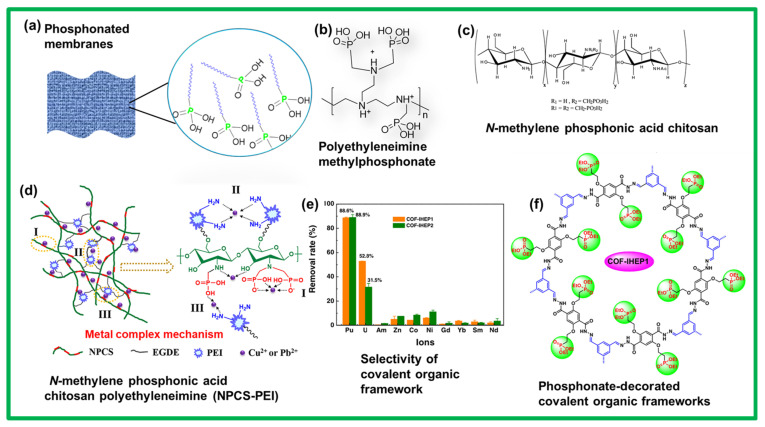
(**a**) Artistic view of a representative membrane or water-purification system based on polymers containing phosphonic acid groups. (**b**) Chemical structure of an ionic polymer used for removing metals from water. (**c**,**d**) Biopolymer (functionalized chitosan)-based assemblies for eliminating metals from water. (**e**) Metal-ion adsorption and selectivity of a (**f**) phosphonated covalent organic framework with well-defined pores and a programed architecture. Adapted with permission from *Carbohydrate Polymers* [102], Copyright 2021 Elsevier Ltd., and [106], Copyright 2019 CCS Chem.

Similar to the sulfonated polymers, their counter derivatives based on phosphonic acids have also been reported in the literature for various applications in coatings, sensors, actuators, and fuel cells [98,99]. Several classes of polymers containing phosphonic acid groups, such as polyarylenes, polyimides, organic–inorganic hybrid polymers, polystyrene, and di- and tri-block copolymers, were synthesized and modified for the preparation of membrane fuel cells. The phosphonic acid group is amphoteric, and thus, acts as a proton donor and acceptor [98]. In phosphonated polymers, the local mobility of the amphoteric groups is essential for the structural diffusion of protons or ions to improve the ion-exchange capacity. In addition, upon interacting with a metallic ion, the phosphonic acid group can undergo two different mechanisms involving electrostatic forces and coordination bonds, depending on the pH of the media [99]. Owing to their pH-dependent metal removal mechanism, phosphonic-acid-based polymers have been extensively investigated for water treatment. Notably, at a pH of 2, phosphonic acid groups exhibit electrostatic interactions with metallic ions; thus, metal-ion exchange with protons is inhibited. When cation exchange with the protons of the phosphonic acid groups is reduced, the selectivity of the acidic group toward certain metallic ions becomes favorable. At above pH = 7, when two phosphonic acid protons are generated, chelation and polyelectrolyte phenomena occur. In addition, phosphonic acid groups display various modes of complexation with metallic ions [100]. Therefore, phosphonated polymers are superior to sulfonated polymers in terms of metal adsorption and selectivity. Figure 5 depicts phosphonic-acid-containing polymers designed for membrane fabrication and water treatment. These materials include artificial and bio-based polymers with programmed morphologies and tunable water-purification efficiencies. Polyethyleneimine (PEI), a well-known synthetic, water-soluble, and nontoxic polymer, was utilized for gene delivery [101]. Owing to its ionic nature, PEI was also used for water treatment. A selective example of a water-treatment membrane based on PEI is shown in Figure 5b. In this example, water-soluble phosphonated PEI was synthesized and tested against U(VI) and Pu/Th(IV) contamination in water [102]. The reported phosphonated PEI architectures exhibited a high loading capacity (0.56–0.80) for uranium. Other phosphonated derivatives of polymers, such as polystyrene, have also been reported for similar applications. However, the major drawback of these polymers is the challenge in processing them into commercially acceptable forms. The challenge in using environmentally friendly materials extends the focus to the usage of bio-based polymers that can be functionalized with phosphonic acid groups. In this case, chitosan, a polysaccharide, with phosphonic acid groups was introduced and explored as a chelating agent for removing metals from water (Figure 5c,d) [103,104,105]. In 2021, an aerogel based on N-methylene phosphonic chitosan was proposed to capture Cu^2+^ and Pb^2+^ from aqueous environments (Figure 5d) [103,104,105]. These functionalized chitosan aerogels with crosslinked architectures were examined for the adsorption of Cu^2+^ and Pb^2+^ at different pH values. The findings reveal that these aerogels could be used as efficient adsorbents for heavy-metal-ion removal from water. In another study, a composite of chitosan/cellulose phosphonate was prepared to form a hydrogel for the ultrafast and efficient removal of Pb(II) and Cu(II) from wastewater [105]. The experimental results showed the high selectivity, excellent adsorption capacity, and ultrafast adsorption rate of chitosan/cellulose phosphonate against Pb(II) and Cu(II); thus, this composite is an excellent bio-based adsorbent or hydrogel for water purification [105].

In 2023, using phosphonated cellulose in combination with PEI resulted in new cellulose-based materials with improved adsorption abilities to remove toxic Pb(II) and Cu(II) from water in a short time [106]. These experimental results indicate that acid-functionalized bio-based polymers will contribute toward manufacturing water-purification systems or assemblies in an environmentally friendly manner.

Further exploration has included other interesting polymers known as COFs, as described in the previous section. In this study, two types of COFs with phosphonic acid groups were synthesized in controlled and rational modes (Figure 5f) [106]. These phosphonated COFs evolved into two-dimensional nanosheets that structurally facilitated water permeation through the interlayer spaces. The phosphonic acid groups present in the skeleton of the COFs acted as chelating sites for U(VI) and Pu(IV). These highly stable phosphonated COFs showed remarkable selectivity and uptake for U(VI) and Pu(IV), thereby efficiently removing radioactive metals from water (Figure 5e) [107].

In contrast, the phosphonic acid group (-PO_3_H_2_) can bind with a number of metallic ions via various combinations or modes due to the presence of two –OH groups and a separate oxygen atom. There are around 18 possible coordination modes that a phosphonic acid group can undergo. These modes are affected by the nature of the metallic ion, pH, temperature, and any substituents linked in the vicinity of the phosphonic acid group. As compared with its sulfonic acid counterpart, the phosphonic acid forms a stable bond with the respective metal, and hence, can be superior for metal adsorption in water treatment. Additionally, the presence of two deprotonation sites in phosphonic acid offers effective metal interactions and control over the release of the metallic ions. Furthermore, the configuration of phosphonic acid groups in a polymeric network defines the formation of porous structure and interconnected channels with perforation, in which polluted water can pass through and allow the metallic ions to interact with the acidic groups in a timely fashion. Usually, the phosphonic-acid-based polymer networks express a rigid and well-preserved network of hydrogen bonds, which also provide further stability to the membrane. However, at a certain temperature (>90 °C), the porosity within the polymeric network can be disrupted due to condensation water molecules produced from the –OH groups of the acid. This condensation or anhydride formation during operation at a high temperature causes the phosphonic-acid-based membranes to suffer a reduction in stability, as well as performance.

## 5. Polymers Functionalized with Carboxylic Acid Groups for Water Purification

**Figure 6:**
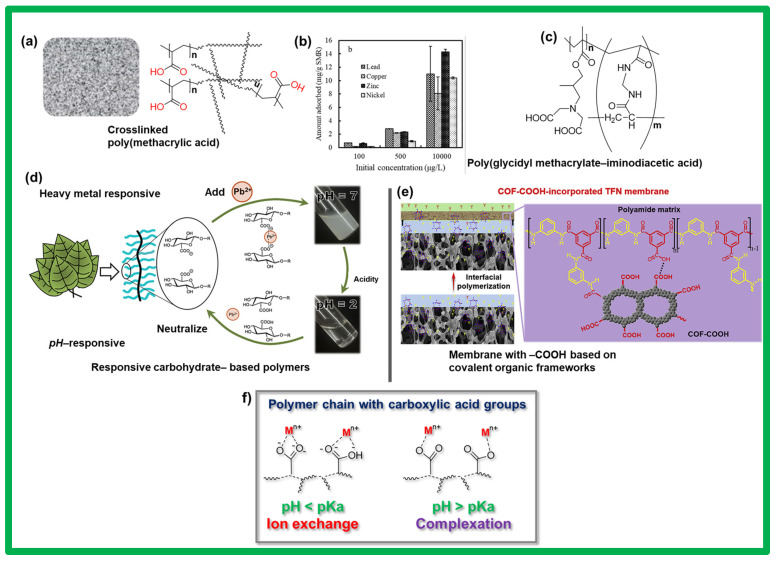
Examples of a few polymers containing major carboxylic acid groups and covalent organic frameworks for water purification. (**a**) Crosslinked polyacrylic system with an artistic picture of the membrane, and (**b**) metal-ion adsorption tests on a crosslinked membrane. (**c**) Chemical structure of crosslinked resin consisting of poly(iminodiacetic acid) and poly(glycidyl methacrylate). (**d**) Carbohydrate-based pH-responsive membrane for water purification, and (**e**) carboxylated, precisely functionalized, and ordered covalent organic frameworks with a porous network of nanochannels. (**f**) Various modes of interaction between the metal and carboxylic acid group. Adapted with permission from the *Journal of Environmental Sciences* [111], Copyright 2018 Elsevier Ltd.; *ACS Cent. Sci.* [117], Copyright 2024, American Chemical Society; and *Journal of Membrane Science* [118], Copyright 2020 Elsevier B.V.

For carboxylic-acid-containing polymers, the criteria for selecting a particular acidic group for metal sorption and selectivity must be discussed. Before an acidic polymer precursor is designed for a membrane, the desired application, metallic ions, and pH of the wastewater must be considered as decisive factors for a specific acidic group. While sulfonated and phosphonated polymers were employed to eliminate a high quantity of metal ions with favorable selectivity, the carboxylate polymeric derivatives showed rather distinct behavior when operating at high and low pH values [108,109,110,111]. The carboxylic acid group, as a monoacid, can be negatively charged at high pH levels and reappear uncharged at low pH levels; therefore, it can participate in both ion exchange and complexation (Figure 6f) [109,110,111]. This peculiar nature of carboxylic acid groups has allowed scientists to explore polymers decorated with similar functionalities for water-treatment applications. A group of carboxylic-acid-containing polymers and their related functions, such as metal adsorption, are shown in Figure 6. Polymers based on acrylic acid groups were explored to remove various metallic ions from wastewater. In a series of poly(acrylic acid)-based materials for water treatment, submicron ion-exchange resins were recently designed to remove toxic metals, such as lead, copper, zinc, and nickel, in the presence of natural organic matter [112]. A peculiar feature of these resins is that they can remove metals while leaving natural organic matter in river water. Figure 6a shows a description of these submicron ion-exchange resins and their metal-binding properties. The acrylic-acid-based resins were tested for their ability to remove lead, copper, zinc, and nickel from river water and wastewater. The results revealed a promising performance, with up to 85.9% ± 0.1% removal of lead (Figure 6b).

Further advancements related to polymers containing carboxylic acid groups have expanded to the production of a wide variety of more or less complex polymeric structures composed of different or combinations of co-monomers, such as maleic acid, styrene, salicylic acid, and ethylenediaminetetraacetic acid, to enhance the selectivity [113,114,115,116]. Polymers bearing water-soluble and water-insoluble carboxylic acid groups were also synthesized and tested to successfully remove Fe^3+^, Zn^2+^, Cd^2+^, and Pb^2+^. A crosslinked resin consisting of poly(iminodiacetic acid) and poly(glycidyl methacrylate) was explored as a chelating agent for the removal of heavy metals from wastewater (Figure 6c) [117]. As the focus has shifted toward bio-based polymers, carbohydrate-based polymers with amphiphilic glucuronate side chains capable of selectively binding heavy-metal cations in mixed media were also reported in 2024 (Figure 6d) [118]. These carbohydrate derivatives display responsiveness toward pH levels and can precipitate upon complexation with a metal; hence, they exhibit efficient filtration, with a 99% removal rate for the toxic species (Pb^2+^) from the solution within minutes (Figure 6d). These carbohydrate-based systems are recyclable and can inspire researchers to develop more advanced bio-based materials that are stimulus-responsive and reusable [118]. As highlighted in the previous sections, crystalline and special polymers, namely, COFs, offer further opportunities to develop efficient water-purifying assemblies as membranes owing to their high porosity and well-organized channel structures. Thin-film nanocomposite membranes fabricated from hydrophilic carboxylated COFs (COF–COOH) were recently introduced and explored (Figure 6e) [119]. These COF–COOH membranes contained precise degrees and locations of acidic groups with controlled architectures and porous morphologies; thus, they were highly effective in water desalination and purification (Figure 6e). Other interesting examples of membranes composed of carboxyl-functionalized COFs include a new type of hydrophilic 2D COFs incorporated into polyacrylonitrile (PAN) polymer matrix membranes [120]. These successful attempts have further advanced the fabrication of membranes using acidic-group-containing polymers for water purification. The aforementioned acid-functionalized polymers paved the way for the development of efficient separation or purification membranes for the filtration and adsorption of metals. Thus far, using acidic-polymer-based membranes is regarded as the most efficient and cost-effective water-treatment method. However, research and technology related to these acid-functionalized polymers are still undergoing further refinement to address the existing challenges, which are discussed in the subsequent section.

Among these major acidic functionalities and their respective materials reviewed in here, the carboxylate derivatives were synthesized and used extensively, including for water treatment. Owing to the strong coordination ability and flexible mode of complexation, the carboxylates ligands are the best choice to produce the polymers based on them. The rather symmetrical configuration of the carboxylic acid group can generate a network of hydrogen bonds within a polymer, where the pores can be available selectively to the particular metallic ions and thereby not allowing other counter ions to overcrowd. The most distinctive features of carboxylate ligands or an active monomer include their ability to be monodentate and bidentate as favorable for different coordination modes. Also, in carboxylates, the oxygen atoms can be tri-coordinated, and hence, can participate in tuning the porosity of the resulting polymeric network. While aliphatic carboxylated monomers can add a significant conformational freedom in their relevant polymers, the aromatic ones are also suitable for creating additional rigidity and stability in a membrane. Obviously, due to their C-C chemistry and different symmetrical and unsymmetrical conformations, the carboxylic-acid-based polymers have garnered further attention for more exploration regarding water-purifying polymers.

## 6. Conclusions

Advancements in human society and the associated demands have had an immense impact on the environment. Such negative impacts on the environment are further seen in the form of pollution, such as water pollution. Both rivers and groundwater are polluted to a large extent, causing several terminal diseases in animals and humans. As the problem of polluted water is exacerbated, scientists are exploring and suggesting multiple solutions for water treatment and purification. The technology used to purify waste or polluted water includes the usage of a membrane as a filtration medium. These membranes and related products are based on polymers and composites. Numerous polymers, such as polyethylene terephthalate, PAN, polyvinylidene fluoride, polyethersulfone, polysulfone, polyetheretherketone, PA, polyurethane, and poly(styrene), were fabricated as membranes or films for integration with water-filtering devices. The focus when evaluating the impact of technology on the environment has shifted from artificially produced polymers to bio-based macromolecules, such as cellulose, chitosan, alginate, and other carbohydrate-based polymers, for similar applications. These polymers are functionalized with acidic groups, such as sulfonic, phosphonic, and carboxylic acids, to be used as membranes in water-treatment systems. Such acidic functionalization can be achieved via either monomer polymerization or the post-modification of a particular polymer. Polymers that are programmable and tunable and have a well-defined crystalline structure known as COFs were also explored as alternative materials of choice for membrane fabrication and similar applications. The acidic nature and dissociation of acidic groups play important roles in the removal of toxic metals from wastewater. Although all acid-group-decorated polymers efficiently eliminate metals, their selectivity toward a metallic ion may vary depending on the acidity of these groups. Other features of a membrane, such as the porosity, morphology, thickness, and durability, are also crucial for its operation. Typically, acidic polymers are processed by using methods such as IP, sputtering, solution casting, extrusion, melt pressing, phase inversion, and electrospinning. Acidic polymers, including their derivatives and composites, represent considerable progress in water purification. Some of these acidic polymers endowed with a peculiar functionality, for example, to respond to a change in the pH or temperature and perform accordingly, have been successfully integrated into technology. Recyclability and regeneration of these acidic polymers are of great interest. More attention is now being paid to the usage of biodegradable polymers to functionalize them with acidic groups and reduce their environmental impact. In conclusion, the advancements in acidic polymers have significantly contributed toward innovation and technology related to water-treatment systems. Nevertheless, the quest to create advanced polymers that exhibit improved performance and recyclability has not ended.

## 7. Future Research and Technological Challenges

**Figure 7:**
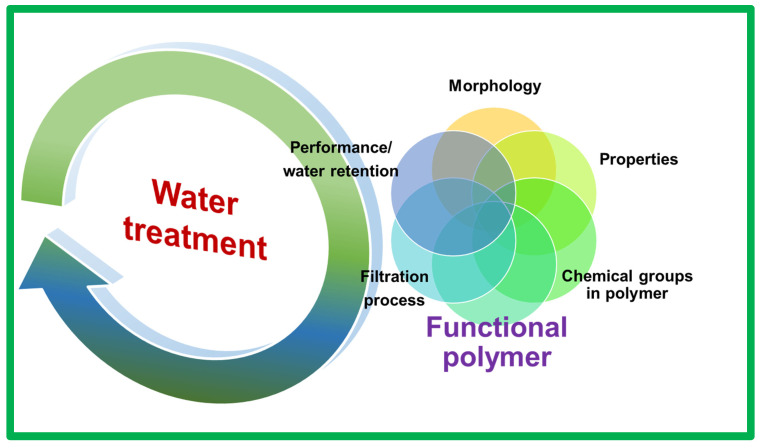
Correlations between the various properties and functions of an acidic-group-bearing polymer fabricated as a membrane for water treatment. These properties must not be compromised when designing a new polymer. Therefore, further advancements are still needed to obtain a polymeric system, in which all the properties are combined in a holistic approach to achieve the highest performance for a water-filtering device.

Although some acidic polymeric membranes are commercially utilized, certain challenges can limit their usage, and thus, need to be addressed. Figure 7 presents the challenges that require broad attention and solutions in a holistic approach. One of the major issues in these acid-based membranes is their extreme water uptake, which causes them to swell and reduces their performance and durability. The water-uptake ability is directly related to the acid groups and hydrophilicity of the original polymer used in the fabrication. Therefore, the degree of acid functionality and the balance between the water uptake and hydrophilicity of the polymer must be tuned prior to production. In addition, the acid groups and chemical nature of the polymer must meet the requirements of the processing method. Because the morphology with well-defined pores and functional channels is essential in the membrane to ensure appropriate performance, an optimized processing step in which such features of the membrane are not compromised must be adopted. Further property enhancements in these polymers with regard to their pores, high-loading-metal capacity, water retention, and stability are recommended by accessing more “polymeric networks”, in which the direction of the channels (hydrogen bonded network of acid groups) is guided in such a way that the factors such as pressure and temperature can also influence the flow and adsorption of the metallic ions. A suitable polymer exhibiting high efficiency is still required for membrane fabrication to facilitate smooth operation under various operational parameters, such as the pH, temperature, pressure, or any other atmospheric obstacle. Therefore, more advanced and responsive polymers must be developed and tested. In addition, the cost associated with the production of acid-containing polymers must be reduced. To reduce the emissions of greenhouse gases resulting from the synthesis of raw materials, innovative routes for manufacturing similar active polymers can also be implemented.

The chemistry of the acid derivatives of COFs must be further modified to obtain processable COFs that exhibit enhanced water-purification performance. Although acid-group-functionalized COFs have a high precision because of the number of acidic groups, predesigned morphology, and tunable pores, their stability must be enhanced for technological integration.

## Figures and Tables

**Table 1:** Various functionalized polymers with different acidic functionalities and their metal-binding properties for water treatment or purification.

No.	Ionic Polymer/Polyelectrolyte	Product	Metal Binding	Applications	Ref.
1.	Poly(sodium 4-styrene sulfonate-*co*-acrylic acid)	Water-insoluble, crosslinked resin	Hg(II),Cd(II), Cr(III), Zn(II), and Pb(II)	Water treatment	[21,22]
2.	Sulfonated poly(glycidyl methacrylate)	Resin nanoparticles	Cd^2+^	Water treatment	[23]
3.	Copolymer of sodium styrene sulfonate	Water insoluble resins	Hg(II)	Water treatment	[24]
4.	Sulfonated polyethyleneimine	Crosslinked polymer	Hg up to 87%	Filters for household taps	[25]
5.	Sulfonated/phosphonated polystyrene	Gel resins	>99% selective for Eu(III)	Metal-ion separation	[26]
6.	Polyethyleneiminemethylene phosphonic acid	Extractant polymer	Cu(II)	Solid-phase extraction	[27]
7.	Styrene−divinylbenzene phosphonated copolymer	Chelating resins	Ca^2+^, Cu^2+^, and Ni^2+^	Wastewater treatment	[28]
8.	Poly(glycidyl methacrylate)/silicon dioxide–iminodiacetic acid	Composite chelating materials	Pb^2+^	Metal separation	[29]
9.	Poly(acrylic acid) sodium salt	Polyelectrolyte membrane	Cu(II)	Wastewater treatment	[30]
10.	Polyethylene terephthalate	Capillary-channeled polymer (C-CP) fibers	Cu^2+^, Cu^+^, Ni^2+^, Fe^3+^, and UO_2_^2+^	Solid-phase extraction	[31,32]

**Table 2:** Bio-based acidic polymers with carboxylic, sulfonic, and phosphonic acid functionalities and their target metal for water purification.

No.	Ionic Biopolymers	Product	Metal Binding	Applications	Ref.
1.	Alginate with carboxylic acid groups	Beads/ membranes	Cu, Cr, Ni, and Cd	Water purification	[54]
2.	Poly-γ-glutamic acid	Water-soluble nanoparticles	Cu, Fe, Cd, Ni, Mn, and Pb	Water treatment	[55,56]
3.	Chitosan with carboxylic acid groups	Beads/membrane/hydrogels	Cu, Hg, Pb, Mn, Ni, Co, and Cd	Industrial wastewater treatment	[57,58]
4.	Phosphonated alginate	Nanoparticles/films	Zn, Cu, Pb, Cd, and Fe	-	[59,60]
5.	Sulfonated chitosan	Hydrogels/ microspheres	Cd, Cr, and Zn	-	[61,62]
6.	Phosphonated chitosan	-	Zn, Cu, Pb, Cd, and Fe	-	[63]

## Abbreviations

COFs, covalent organic frameworks; S-CONs, sulfonated COF nanosheets; PA, Polyamide; PBDI, Poly(2,2′-disulfonyl-4,4′-benzidine isophthalamide); PEI, Polyethyleneimine; PAN, Polyacrylonitrile.

## References

[1] Onorato J., Pakhnyuk V., Luscombe C.K. (2016). Structure and design of polymers for durable, stretchable organic electronics. Polym. J..

[2] Sun H.S., Chiu Y.C., Chen W.C. (2017). Renewable polymeric materials for electronic applications. Polym. J..

[3] Liu Z., Ma Z., Qian B., Chan A.Y.H., Wang X., Liu Y., Xin J.H. (2021). A Facile and Scalable Method of Fabrication of Large-Area Ultrathin Graphene Oxide Nanofiltration Membrane. ACS Nano.

[4] Sarkar A.K., Bediako J.K., Choi J.W., Yun Y.S. (2019). Functionalized magnetic biopolymeric graphene oxide with outstanding performance in water purification. NPG Asia Mater..

[5] Abbo H.S., Gupta K.C., Khaligh N.G., Titinchi S.J.J. (2021). Carbon Nanomaterials for Wastewater Treatment. ChemBioEng.

[6] Adeleye A.S., Conway J.R., Garner K., Huang Y., Su Y., Keller A.A. (2016). Engineered nanomaterials for water treatment and remediation: Costs, benefits, and applicability. Chem. Eng. J..

[7] Miyake J., Miyatake K. (2017). Fluorine-free sulfonated aromatic polymers as proton exchange membranes. Polym. J..

[8] Maiti P., Kumar S. (2023). Review on Functional Electrolyte, Redox Polymers, and Solar Conversions in 3G Emerging Photovoltaic Technologies: Progress and Outlook. Energy Fuels.

[9] Jagur-Grodzinski J. (1999). Biomedical application of functional polymers. React. Funct. Polym..

[10] Seppala J., van Bochove B., Lendlein A. (2020). Developing Advanced Functional Polymers for Biomedical Applications. Biomacromolecules.

[11] Potaufeux J.E., Odent J., Notta-Cuvier D., Lauro F., Raquez J.M. (2020). A comprehensive review of the structures and properties of ionic polymeric materials. Polym. Chem..

[12] Wen J., Zhou L., Ye T. (2024). Polymer ionogels and their application in flexible ionic devices. SmartMat.

[13] Hickner M.A. (2010). Ion-containing polymers: New energy & clean water. Mater. Today.

[14] Du C., Ma X., Li J., Wu C. (2017). Improving the charged and antifouling properties of PVDF ultrafiltration membranes by blending with polymerized ionic liquid copolymer P(MMA-b-MEBIm-Br). J. Appl. Polym. Sci..

[15] Song Y., Phipps J., Zhu C., Ma S. (2023). Porous Materials for Water Purification. Angew. Chem..

[16] Xu X., Yang Y., Liu T., Chu B. (2022). Cost-effective polymer-based membranes for drinking water purification. Giant.

[17] Mecerreyes D. (2011). Polymeric ionic liquids: Broadening the properties and applications of polyelectrolytes. Prog. Polym. Sci..

[18] Khomein P., Ketelaars W., Lap T., Liu G. (2021). Sulfonated aromatic polymer as a future proton exchange membrane: A review of sulfonation and crosslinking methods. Renew. Sustain. Energy Rev..

[19] Jang S., Kim S.Y., Jung H.Y., Park M.J. (2018). Phosphonated Polymers with Fine-Tuned Ion Clustering Behavior: Toward Efficient Proton Conductors. Macromolecules.

[20] Kurapati R., Natarajan U. (2023). Role of concentration and hydrophobic nature of weak polyelectrolytes on adsorption structure and thermodynamics at oil-water interface: Study of several carboxylate polymers. Polymer.

[21] Rivas B.L., Muñoz C. (2009). Synthesis and metal ion adsorption properties of poly(4-sodium styrene sulfonate-co-acrylic acid). J. Appl. Polym. Sci..

[22] Ji C., Qu R., Sun C., Wang C., Xu Q., Sun Y., Li C., Guo S. (2007). Macroporous chelating resins incorporating heterocyclic functional groups via hydrophilic PEG spacer arms. I. Synthesis and characterization. J. Appl. Polym. Sci..

[23] Elkady M.F., Abu-Saied M.A., Rahman A.M.A., Soliman E.A., Elzatahry A.A., Yossef M.E., Eldin M.S.M. (2011). Nano-sulphonated poly (glycidyl methacrylate) cations exchanger for cadmium ions removal: Effects of operating parameters. Desalination.

[24] Wadi V.S., Mittal H., Fosso-Kankeu E., Jena K.J., Alhassan S.M. (2020). Mercury removal by porous sulfur copolymers: Adsorption isotherm and kinetics studies. Colloids Surf. A Physicochem. Eng. Asp..

[25] Saad D.M.G., Cukrowska E.M., Tutu H. (2012). Sulfonated cross-linked polyethylenimine for selective removal of mercury from aqueous solutions. Toxicol. Environ. Chem..

[26] Alexandratos S.D., Natesan S. (1999). Ion-selective polymer-supported reagents: The principle of bifunctionality. Eur. Polym. J..

[27] Abderrahim O., Didi M.A., Moreau B., Villemin D. (2006). A new sorbent for selective separation of metal: Polyethylenimine methylenephosphonic acid. Solvent Extr. Ion. Exch..

[28] Popa A., Davidescu C.M., Negrea P., Ilia G., Katsaros A., Demadis K.D. (2008). Synthesis and characterization of phosphonate ester/phosphonic acid grafted styrene -Divinylbenzene copolymer microbeads and their utility in adsorption of divalent metal ions in aqueous solutions. Ind. Eng. Chem. Res..

[29] Yang L., Li Y., Jin X., Ye Z., Ma X., Wang L., Liu Y. (2011). Synthesis and characterization of a series of chelating resins containing amino/imino-carboxyl groups and their adsorption behavior for lead in aqueous phase. Chem. Eng. J..

[30] Xiao S.L., Ma H., Shen M.W., Wang S.Y., Huang Q.G., Shi X.Y. (2011). Excellent copper(II) removal using zero-valent iron nanoparticle-immobilized hybrid electrospun polymer nanofibrous mats. Colloid Surf. A.

[31] Panahi H.A., Abdouss M., Ghiabi F., Moniri E., Shoushtari A.M. (2012). Modification and characterization of poly (ethylene terephthalate)-grafted-acrylic acid/acryl amide fiber for removal of lead from human plasma and environmental samples. J. Appl. Polym. Sci..

[32] Pittman J.J., Klep V., Luzinov I., Marcus R.K. (2010). Extraction of metals from aqueous systems employing capillary-channeled polymer (C-CP) fibers modified with poly (acrylic acid) (PAA). Anal. Methods.

[33] Singh K.K., Goel N.K., Kanjilal A., Ruhela R., Kumar V., Bhattacharya K., Tyagi A.K. (2024). Radiation grafted polyacrylic acid–polyurethane foam copolymer for efficient toxic metal removal from aqueous waste: A sustainable approach to waste management. Polym. Bull..

[34] Macarie L., Ilia G. (2010). Poly(vinylphosphonic acid) and its derivatives. Prog. Polym. Sci..

[35] Zhang X., Zhao Y., Xu S., Yang Y., Liu J., Wei Y., Yang Q. (2014). Polystyrene sulphonic acid resins with enhanced acid strength via macromolecular self-assembly within confined nanospace. Nat. Commun..

[36] Markova D., Kumar A., Klapper M., Mullen K. (2009). Phosphonic acid-containing homo-, AB and BAB block copolymers via ATRP designed for fuel cell applications. Polymer.

[37] Kumar A., Pisula W., Markova D., Klapper M., Mullen K. (2012). Proton-Conducting Poly(phenylene oxide)–Poly(vinyl benzyl phosphonic acid) Block Copolymers via Atom Transfer Radical Polymerization. Macromol. Chem. Phys..

[38] Kumar A. (2020). Cooperative proton conduction in sulfonated and phosphonated hybrid random copolymers. J. Mater. Chem. A.

[39] Jaffe H.H., Freedman L.D., Doak G.O. (1953). The Acid Dissociation Constants of Aromatic Phosphonic Acids. I. Meta and Para Substituted Compounds. J. Am. Chem. Soc..

[40] Sata T., Yoshida T., Matsusaki K. (1996). Transport properties of phosphonic acid and sulfonic acid cation exchange membranes. J. Membr. Sci..

[41] Waresindo W.X., Luthfianti H.R., Priyanto A., Hapidin D.A., Edikresnha D., Aimon A.H., Suciati T., Khairurrijal K. (2023). Freeze–thaw hydrogel fabrication method: Basic principles, synthesis parameters, properties, and biomedical applications. Mater. Res. Express.

[42] Raps D., Hossieny N., Park C.B., Altstadt V. (2015). Past and present developments in polymer bead foams and bead foaming technology. Polymer.

[43] Palit S., Kreplak L., Frampton J.P. (2022). Formation of Core-Sheath Polymer Fibers by Free Surface Spinning of Aqueous Two-Phase Systems. Langmuir.

[44] Bacheller S., Dianat G., Gupta M. (2021). Synthesis of pH-Responsive Polymer Sponge Coatings and Freestanding Films via Vapor-Phase Deposition. ACS Appl. Polym. Mater..

[45] Sibiryakov B., Leite L.W.B., Sibiriakov E. (2021). Porosity, specific surface area and permeability in porous media. J. Appl. Geophys..

[46] Kiefer J., Rasul N.H., Ghosh P.K., von Lieres E. (2014). Surface and bulk porosity mapping of polymer membranes using infrared spectroscopy. J. Membr. Sci..

[47] Léniz-Pizarro F., Rudel H.E., Briot N.J., Zimmerman J.B., Bhattacharyya D. (2023). Membrane Functionalization Approaches toward Per- and Polyfluoroalkyl Substances and Selected Metal Ion Separations. ACS Appl. Mater. Interfaces.

[48] Li G., Chan Yao C., Wang J., Xu Y. (2017). Synthesis of tunable porosity of fluorine-enriched porous organic polymer materials with excellent CO_2_, CH_4_ and iodine adsorption. Sci. Rep..

[49] Soldatov V., Pristavko S., Zelenkovskii V., Kosandrovich E. (2013). Hydration of ion exchangers: Thermodynamics and quantum chemistry calculations. React. Funct. Polym..

[50] Pearson R.G. (1963). Hard and Soft Acids and Bases. J. Am. Chem. Soc..

[51] Yolsal U., Horton T.A.R., Wang M., Shaver M.P. (2020). Polymer-supported Lewis acids and bases: Synthesis and applications. Prog. Polym. Sci..

[52] Shi D., Ran M., Zhang L., Huang H., Li X., Chen M., Akashi M. (2016). Fabrication of Biobased Polyelectrolyte Capsules and Their Application for Glucose-Triggered Insulin Delivery. ACS Appl. Mater. Interfaces.

[53] Dong Z., Wu J., Shen X., Hua Z., Liu G. (2023). Bioinspired nucleobase-containing polyelectrolytes as robust and tunable adhesives by balancing the adhesive and cohesive properties. Chem. Sci..

[54] Yang L., Guo J., Wu J., Yang Y., Zhang S., Song J., An Q., Gong Y. (2017). Preparation and properties of a thin membrane based on sodium alginate grafting acrylonitrile. RSC Adv..

[55] Syeda H.I., Muthukumaran S., Baskaran K. (2023). Polyglutamic acid and its derivatives as multi-functional biopolymers for the removal of heavy metals from water: A review. J. Water Process Eng..

[56] Sakamoto S., Kawase Y. (2016). Adsorption capacities of poly-γ-glutamic acid and its sodium salt for cesium removal from radioactive wastewaters. J. Environ. Radioact..

[57] Boamah P.O., Huang Y., Hua M., Zhang Q., Wu J., Onumah J., Sam-Amoah L.K., Boamah P.O. (2015). Sorption of heavy metal ions onto carboxylate chitosan derivatives—A mini-review. Ecotoxicol. Environ. Saf..

[58] Sun Y., Chen A., Pan S.Y., Sun W., Zhu C., Shah K.J., Zheng H. (2019). Novel chitosan-based flocculants for chromium and nickle removal in wastewater via integrated chelation and flocculation. J. Environ. Manag..

[59] Jayakumar R., Rajkumar M., Freitas H., Selvamurugan N., Nair S.V., Furuike T., Tamura H. (2009). Preparation, characterization, bioactive and metal uptake studies of alginate/phosphorylated chitin blend films. Int. J. Biol. Macromol..

[60] Duan J., Li W., Wei Y. (2024). Fabrication of sodium alginate doped phosphoric acid composite hydrogel and its application of the adsorption of La (III) in wastewater. J. Chromatogr. A.

[61] Weltrowski M., Martel B., Morcellet M. (1996). Chitosan *N*-benzyl sulfonate derivatives as sorbents for removal of metal ions in an acidic medium. J. Appl. Polym. Sci..

[62] Gu F., Geng J., Li M., Chang J., Cui Y. (2019). Synthesis of Chitosan–Ignosulfonate Composite as an Adsorbent for Dyes and Metal Ions Removal from Wastewater. ACS Omega.

[63] Mady M.F., Abdel-Azeim S., Kelland M.A. (2021). Investigation of the Antiscaling Performance of Phosphonated Chitosan for Upstream Petroleum Industry Application. ACS Sustain. Chem. Eng..

[64] Cheng S., Grest G.S. (2016). Dispersing Nanoparticles in a Polymer Film via Solvent Evaporation. ACS Macro Lett..

[65] Kravchenko V.S., Potemkin I.I. (2020). Nanodroplets of Polymer Solutions on Solid Surfaces: Equilibrium Structures and Solvent Evaporation. Macromolecules.

[66] Rao J.P., Geckeler K.E. (2011). Polymer nanoparticles: Preparation techniques and size-control parameters. Prog. Polym. Sci..

[67] Lu T.D., Wang Q., Gu S.S., Sun S.P. (2023). Beyond Symmetry: Exploring Asymmetric Electrospun Nanofiber Membranes for Liquid Separation. Adv. Funct. Mater..

[68] Bachler S., Ort M., Kramer S.D., Dittrich P.S. (2021). Permeation Studies across Symmetric and Asymmetric Membranes in Microdroplet Arrays. Anal. Chem..

[69] Nielen W.M., Willot J.D., de Vos W.M. (2020). Aqueous Phase Separation of Responsive Copolymers for Sustainable and Mechanically Stable Membranes. ACS Appl. Polym. Mater..

[70] Clarizia G., Tasselli F., Simari C., Nicotera I., Bernardo P. (2019). Solution Casting Blending: An Effective Way for Tailoring Gas Transport and Mechanical Properties of Poly(vinyl butyral) and Pebax2533. J. Phys. Chem. C.

[71] Raje A., Koll J., Schneider E.S., Gerogopanos P. (2023). A novel organic solvent-free method for manufacturing polyethersulfone hollow fiber membranes using melt extrusion. J. Membr. Sci..

[72] Tekin F.S., Zeynep P., Emecen C. (2023). Controlling Cellulose Membrane Performance via Solvent Choice during Precursor Membrane Formation. ACS Appl. Polym. Mater..

[73] Lavielle N., Hebraud A., Schlatter G., Meyer L.T., Rossi R.M., Popa A.M. (2013). Simultaneous Electrospinning and Electrospraying: A Straightforward Approach for Fabricating Hierarchically Structured Composite Membranes. ACS Appl. Mater. Interfaces.

[74] Avossa J., Herwig G., Toncelli C., Itel F., Rossi R.M. (2022). Electrospinning based on benign solvents: Current definitions, implications and strategies. Green Chem..

[75] Cha B.J., Yang J.M. (2007). Preparation of poly(vinylidene fluoride) hollow fiber membranes for microfiltration using modified TIPS process. J. Membr. Sci..

[76] Chandavasu C., Xanthos M., Sirkar K.K., Gogos C.G. (2003). Fabrication of microporous polymeric membranes by melt processing of immiscible blends. J. Membr. Sci..

[77] Frenot A., Chronakis I.S. (2003). Polymer nanofibers assembled by electrospinning. Curr. Opin. Colloid Interface Sci..

[78] Higashihara T., Matsumoto K., Ueda M. (2009). Sulfonated aromatic hydrocarbon polymers as proton exchange membranes for fuel cells. Polymer.

[79] Adamski M., Peressin N., Holdcroft S. (2021). On the evolution of sulfonated polyphenylenes as proton exchange membranes for fuel cells. Mater. Adv..

[80] Gioacchino M.D., Fabio Bruni F., Imberti S., Ricci M.A. (2020). Hydration of Carboxyl Groups: A Route toward Molecular Recognition?. J. Phys. Chem. B.

[81] Pereira R.P., Felisberti M.I., Rocco A.M. (2006). Intermolecular interactions and formation of the hydration sphere in phosphonic acid model systems as an approach to the description of vinyl phosphonic acid based polymers. Polymer.

[82] Persson I. (2024). Structure and size of complete hydration shells of metal ions and inorganic anions in aqueous solution. Dalton Trans..

[83] Poźniak G., Bryjak M., Trochimczuk W. (1995). Sulfonated polysulfone membranes with antifouling activity. Die Angew. Makromol. Chem..

[84] Sheela A.S.J.J., Moorthy S., Maria B., Karthikeyan M., Dinakaran S., Kannaiyan P., Deivanayagam P. (2023). Sulfonated Poly Ether Sulfone Membrane Reinforced with Bismuth-Based Organic and Inorganic Additives for Fuel Cells. ACS Omega.

[85] Kruczek B., Matsuura T. (1998). Development and characterization of homogeneous membranes de from high molecular weight sulfonated polyphenylene oxide. J. Membr. Sci..

[86] Farrokhzad H., Kikhavani T., Monnaie F., Ashrafizadeh S.N., Koeckelberghs G., Van Gerven T., Van der Bruggen B. (2015). Novel composite cation exchange films based on sulfonated PVDF for electromembrane separations. J. Membr. Sci..

[87] Bora P., Bhuyan C., Rajguru P., Hazarika S. (2023). A Gemini basic ionic liquid and functionalized cellulose nanocrystal-based mixed matrix membrane for CO_2_/N_2_ separation. Chem. Commun..

[88] Wang X., Feng X., Li Q., Dong Z. (2024). Surface Functionalization Strategy for Cellulose Membranes Based on Silanization and Thiol–Ene Click Chemistry. ACS Appl. Polym. Mater..

[89] Shi S., Zhu K., Chen X., Hu J., Zhang L. (2019). Cross-Linked Cellulose Membranes with Robust Mechanical Property, Self-Adaptive Breathability, and Excellent Biocompatibility. ACS Sustain. Chem. Eng..

[90] Thiangtham S., Saito N., Manuspiya H. (2022). Asymmetric Porous and Highly Hydrophilic Sulfonated Cellulose/Biomembrane Functioning as a Separator in a Lithium-Ion Battery. ACS Appl. Energy Mater..

[91] Lander S., Erlandsson J., Vagin M., Gueskine V., Korhonen L., Berggren M., Wågberg L., Crispin X. (2022). Sulfonated Cellulose Membranes: Physicochemical Properties and Ionic Transport versus Degree of Sulfonation. Adv. Sustain. Syst..

[92] Lee Jin C., Lee S.-Y., So S. (2024). Enhancing Water Absorption in Sulfonated Poly(arylene ether sulfone) Polymer Electrolyte Membranes by Reducing Chain Entanglement through Constrained Deswelling. ACS Appl. Polym. Mater..

[93] Côté A.P., Shimizu G.K. (2003). The supramolecular chemistry of the sulfonate group in extended solids. Coord. Chem. Rev..

[94] Geng K., He T., Liu R., Dalapati S., Tian Tan K., Li Z., Tao S., Gong Y., Jiang Q., Jiang D. (2020). Covalent Organic Frameworks: Design, Synthesis, and Functions. Chem. Rev..

[95] Meng W., Xue Q., Zhu J., Zhang K. (2024). Exploiting sulfonated covalent organic frameworks to fabricate long-lasting stability and chlorine-resistant thin-film nanocomposite nanofiltration membrane. NPJ Clean Water.

[96] Khodabakhshi M.R., Goodarzi V. (2021). Preparing and analysis an innovative membrane based on polyethersulfone/sulfonated polyethersulfone/organically modified nanoclay: Ability to heavy metal removal. Mater. Today Commun..

[97] Fraser A.C., Yankey J., Coronell O., Dingemans T. (2023). A Sulfonated All-Aromatic Polyamide for Heavy Metal Capture: A Model Study with Pb(II). ACS Appl. Polym. Mater..

[98] Solimando X., Catel Y., Moszner N., Robin J.J., Monge S. (2020). Smart functionalized phosphonic acid based copolymers: New structures for old purposes. Polym. Chem..

[99] Rathore K., Jangir R. (2024). Insight into Synthesis, properties and applications of metal Phosphonates: Emphasis on catalytic activities. Inorganica Chim. Acta.

[100] Lacour S., Deluchat V., Bollinger J.C., Serpaud B. (1998). Complexation of trivalent cations (Al(III), Cr(III), Fe(III)) with two phosphonic acids in the pH range of fresh waters. Talanta.

[101] Wiseman J.W., Goddard C.A., McLelland D., Colledge W.H. (2003). A comparison of linear and branched polyethylenimine (PEI) with DCChol/DOPE liposomes for gene delivery to epithelial cells In Vitro and In Vivo. Gene Ther..

[102] Lahrouch F., Sofronov O., Creff G., Rossberg A., Henning C., Auwer C.D., Giorgio C.D. (2017). Polyethyleneimine methylphosphonate: Towards the design of a new class of macromolecular actinide chelating agents in the case of human exposition. Dalton Trans..

[103] Liu T., Gou S., He Y., Fang S., Zhou L., Gou G. (2021). N-methylene phosphonic chitosan aerogels for efficient capture of Cu2+ and Pb2+ from aqueous environment. Carbohydr. Polym..

[104] Ramos V.N., Rodriguez N.M., Diaz M.F., Rodriguez M.S., Heras A., Agullo E. (2003). N-methylene phosphonic chitosan. Effect of preparation methods on its properties. Carbohydr. Polym..

[105] Sun J., Hu R., Zhao X., Liu T., Bai Z. (2024). A novel chitosan/cellulose phosphonate composite hydrogel for ultrafast and efficient removal of Pb(II) and Cu(II) from wastewater. Carbohydr. Polym..

[106] Sun J., Zhao X., Hu R., Sun G., Zhao H., Liu W., Nai Z., Jiang X., Cui Y. (2023). Cellulose phosphonate/polyethyleneimine nano-porous composite remove toxic Pb(II) and Cu(II) from water in a short time. Int. J. Biol. Macromol..

[107] Yu J., Yuan L., Wang S., Lan J., Zheng L., Xu C., Chen J., Wang L., Huang Z., Tao W. (2019). Phosphonate-Decorated Covalent Organic Frameworks for Actinide Extraction: A Breakthrough Under Highly Acidic Conditions. CCS Chem..

[108] Jeon C., Park J.Y., Yoo Y.J. (2002). Characteristics of metal removal using carboxylated alginic acid. Water Res..

[109] Pesonen H., Sillanpaa A., Aksela R., Laasonen K. (2005). Density functional complexation study of metal ions with poly(carboxylic acid) ligands. Part 1. Poly(acrylic acid) and poly(α-hydroxy acrylic acid). Polymer.

[110] Tamura T., Uehara H., Ogawara K., Kawauchi S., Satoh M., Komiyama J. (1999). Dissociation behavior of poly(α-hydroxy acrylic acid). J. Polym. Sci. Part. B Polym. Phys..

[111] Seigou K., Toshiaki K., Koichi I., Akira M. (1990). Dissociation behavior of poly(Fumaric acid) and poly (maleic acid). 2. model calculation. Macromolecules.

[112] Murray A., Ormeci B. (2019). Use of polymeric sub-micron ion-exchange resins for removal of lead, copper, zinc, and nickel from natural waters. J. Environ. Sci..

[113] Akperov E.O., Maharramov A.M., Akperov O.G. (2009). Uranyl ion adsorption using novel cross-linked maleic anhydride-allyl propionate-styrene terpolymer. Hydrometallurgy.

[114] Hajiyeva S.R., Bahmanova F.N., Alirzaeva E.N., Shamilov N.T., Chyragov F.M. (2018). Uranium Preconcentration with a Chelating Sorbent Based on Maleic Anhydride–Styrene Copolymer. Radiochemistry.

[115] Panahi H.A., Sadeghi H.B., Farahmandnejad N., Badr A.R., Moniri E. (2012). Removal of cobalt from human serum and environmental samples by adsorption using Amberlite XAD-2-salicylic acid-iminodiacetic acid. Desalin. Water Treat..

[116] Senkal B.F., Bicak N. (2001). Glycidyl methacrylate based polymer resins with diethylene triamine tetra acetic acid functions for efficient removal of Ca(II) and Mg(II). React. Funct. Polym..

[117] Jiang J., Ma X.S., Xu L.Y., Wang L.H., Liu G.Y., Xu Q.F., Lu J.M., Zhang Y. (2015). Applications of chelating resin for heavy metal removal from wastewater. e-Polymers.

[118] Jeon S., Haynie T., Chung S., Callmann C.E. (2024). Bioinspired, Carbohydrate-Containing Polymers Efficiently and Reversibly Sequester Heavy Metals. ACS Cent. Sci..

[119] Xu L., Yang T., Li M., Chang J., Xu J. (2020). Thin-film nanocomposite membrane doped with carboxylated covalent organic frameworks for efficient forward osmosis desalination. J. Membr. Sci..

[120] Duon P.H., Kuehl V.A., Mastorovich B., Hoberg J.O., Parkinson B.A., Li-Oakey K.D. (2019). Carboxyl-functionalized covalent organic framework as a two-dimensional nanofiller for mixed-matrix ultrafiltration membranes. J. Membr. Sci..

